# Recovery of Acute Ophthalmoplegia after Hyaluronic Acid Filler Injections to the Temples: A Case Report and Review of the Literature

**DOI:** 10.1055/s-0043-1761211

**Published:** 2023-03-28

**Authors:** Fatemeh-sadat Tabatabaei, Amirali Azimi, Seyyed Shahabeddin Tabatabaei, Hossein Pakdaman

**Affiliations:** 1 Students' Scientific Research Center (SRCC), School of Medicine, Tehran University of Medical Sciences, Tehran, Iran; 2 Department of Neurology, Shahid Beheshti University of Medical Sciences, Tehran, Iran; 3 Brain Mapping Research Center, Shahid Beheshti University of Medical Sciences, Tehran, Iran

**Keywords:** ophthalmoplegia, surgery, plastic, hyaluronic acid, cosmetics

## Abstract

Soft tissue filler injection is the second most common nonsurgical cosmetic procedure. Despite the safety of fillers, as use has grown, so has the number of patients affected by adverse events. Ophthalmoplegia following cosmetic filler injection is a rare complication, mostly occurring after injection to the glabella, nasolabial fold, periorbital, and lateral nasal site. In all cases where ophthalmoplegia has been reported following fillers, patients have simultaneously experienced vision loss and other ocular symptoms. We report a case of isolated acute ophthalmoplegia following hyaluronic acid injection solely in the temple region. A 40-year-old woman, 3 hours after the procedure, presented to our hospital with left eye ophthalmoplegia, ptosis, and hypotropia. Treatment started with hyaluronidase, steroids, and anticoagulants. After 4 weeks, left eye ophthalmoplegia remained unchanged, and through a 10-week follow-up, all left ocular movements improved, and only mild hypotropia and ptosis persisted. This case report shows that ophthalmoplegia may also happen with temple region filler injections. We also review available prevention techniques and treatments to avoid such complications when performing soft tissue fillers for gaunt appearance correction.

## Introduction

Soft tissue filler injection is the second nonsurgical cosmetic procedure in the United States, following botulinum toxin injection for aesthetic purposes. 1 Fillers are used for tightening loose skin, restoring volume, and correcting wrinkles and folds. Its use in facial rejuvenation has expanded over the past decades. Despite the safety of fillers, their adverse effects have been reported and identified more than before with the increasing use of fillers. Common complications are erythema, edema, localized bruising, filler migration, allergic response, and bumps under the skin to more severe sequels, such as skin necrosis, visual loss, or nerve paralysis, temporary or permanent in some cases. 2

Ocular complications of fillers are mainly attributed to vascular occlusion events. 3 In all cases that ophthalmoplegia has been reported following filler injections, patients have experienced vision loss or other ocular symptoms. To the best of our knowledge, only one article has reported two cases of isolated ophthalmoplegia; in both cases, in addition to the temple area, injections were performed in other face sites associated with ocular complications (periorbital and lateral nasal). 4

This article presents a case of isolated acute ophthalmoplegia following filler injection solely in the temple region.

## Case

A 40-year-old woman with a history of lip filler injection underwent cosmetic hyaluronic acid filler injections to the temples by an experienced physician, through a 27-gauge cannula. First, the filler was injected into the right temporal region with no complications. Then, during injection to the left temple, the patient experienced a sudden frontal headache, severe eye pain, nausea, and vomiting. The procedure was stopped immediately, though the mentioned symptoms remained. Hyaluronidase treatment was given in less than 15 minutes. About 20 minutes after starting the injection, the patient complained of diplopia and was referred to the emergency department for further examination and treatment.

**Fig. 1:**
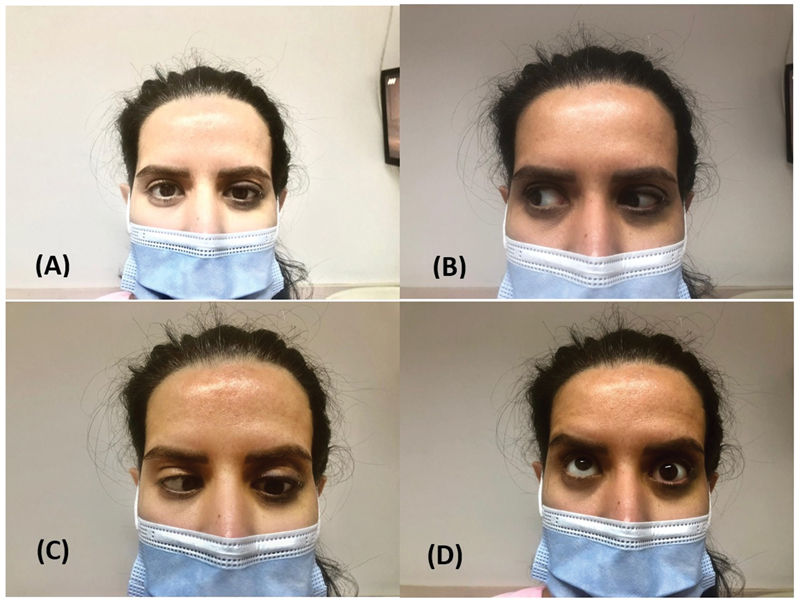
Initial exam of ocular movements. ( **A** ) Mild hypotropia and ptosis. ( **B** ) Right gaze. ( **C** ) Left gaze. ( **D** ) Upward gaze.

We visited the patient 3 hours after the event. The patient had binocular diplopia, frontal headache, eye pain, severe nausea, and vomiting. No vision loss or blurred vision of the left eye was reported. On ocular examinations, pupillary light reflex, ocular tonometry, ophthalmoscopy, visual acuity, and right eye movements were normal. The left eye had mild hypotropia and ptosis. Abduction and supraduction of the left eye were severely impaired; in addition, infraduction, extorsion, and intorsion were mildly limited. Adduction was the only intact left ocular movement ( Fig. 1 ).

**Fig. 2:**
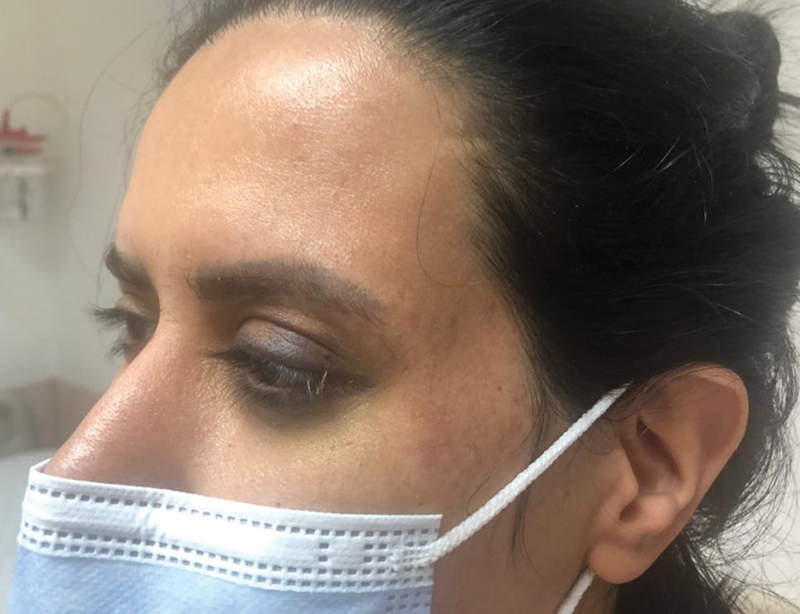
No skin lesion in initial exam.

The rest of neurological examinations, including cranial nerves, were normal. No skin lesions were seen at the injection site ( Fig. 2 ). The patient had no fever, and other vital signs were stable.

Superficial soft tissue ultrasonography of the injection site reported no mass effect or subcutaneous collection. Magnetic resonance imaging (MRI) of the head and orbit, MR angiography, and MR venography of the head were unremarkable. Notably, there were no infarction, hemorrhage, or mass effect findings.

After admission, treatment started with intravenous dexamethasone 8 mg three times daily and subcutaneous injection of enoxaparin 40 mg twice daily empirically for possible ischemic events, which administered for 5 days. Brimonidine eye drops were also prescribed. Enoxaparin 40 mg daily, brimonidine eye drops twice daily plus intravenous ondansetron 4 mg, and acetaminophen 1000 mg if needed, resumed until discharge. Headache, eye pain, nausea, and vomiting resolved a few hours after steroid administration. The patient was discharged after 2 weeks with persistent diplopia.

Through a 4-week follow-up, extensive ophthalmoplegia in the left eye remained unchanged, with involvement of all eye muscles except for the medial rectus.

**Fig. 3:**
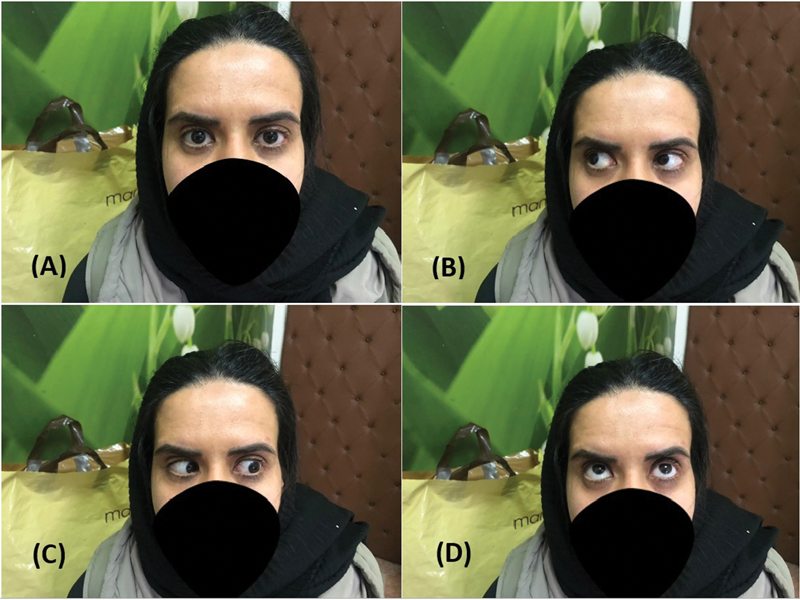
Ocular movements' examination after 10 weeks of fallow-up. ( **A** ) Mild hypotropia and ptosis. ( **B** ) Right gaze. ( **C** ) Left gaze. ( **D** ) Upward gaze.

With a 10-week follow-up, all left ocular movements improved, and only mild hypotropia and ptosis persisted ( Fig. 3 ).

## Discussion

Although hyaluronic acid injection is considered safe for cosmetic purposes, there are reports of ocular complications following injection into the facial skin. Lee et al, in their review on 50 cases of ocular complications after filler injection, found that most ocular complications include visual loss with or without ophthalmoplegia, usually in nasal, glabella, and periorbital injections. 5 A systematic review have found 61 cases with ocular complications after periocular hyaluronic acid injections in an 18-month duration. 6

However, rare cases of blindness have been reported following filler injection into the temporal region, and there are only two cases of isolated ophthalmoplegia following temple region injection. 4 In this report, we presented a case of isolated ophthalmoplegia with no other ocular side effects after filler injection, particularly in the left temple region.

According to recent studies, the most discussed reason for ocular complications is ischemic events due to the possible intra- or periarteriolar injection. The temporal region has complex anatomy due to multiple vessels running in the different tissue layers. The anatomical layers of the temporal region are the skin, subcutaneous tissue, superficial temporal fascia, deep temporal fascia, superficial temporal fat pad, and temporalis muscle. There are several critical vessels located on the margin of the temporal region, including the zygomaticofacial artery (medially) deriving distal to the internal carotid artery, and all terminal branches of the external carotid artery, including the zygomatico-orbital (medially), middle temporal, and the superficial temporal arteries (both laterally). 7 Another important artery which could have been involved in our case is anterior deep temporal artery. Due to anastomoses with the ophthalmic artery, these arteries are of particular concern as injecting into them may result in filler emboli and subsequent blindness.

In this case, the patient had ocular symptoms immediately after the injection, which raises vascular events as the leading cause of the patient's situation. The accepted theory of vascular events proposed in previous studies is the retrograde arterial flow mechanism. During the injection, the needle tip enters the small arterioles, and with excessive pressure on the plunger or long injection time, the pressure inside the needle overcomes the arterial pressure, which causes the retrograde movement of the filler toward the proximal parts of the artery. Then by cutting off the pressure inside the needle, the vascular pressure behind the filler material can move these substances to distal vascular branches parallel to the primarily injected arteriol, also known as embolia cutis medicamentosa (Freudenthal–Nicolau syndrome). 8 Complications have varied according to the possibility of various ophthalmic artery branches or even internal carotid artery involvement, although the initial injection site was the same. Central retinal artery involvement results in vision loss, 9 and the involvement of arterioles supplying the ocular muscles leads to ophthalmoplegia. 10 Since the involvement of the ophthalmic artery occurs more often than the arterioles supplying the ocular muscles, ophthalmoplegia has been reported to be less frequent and simultaneously with vision loss in almost all reported cases. 5

When a large-bore needle and a large syringe are used, the pressure could be enough to drive the dense material into the internal carotid artery, resulting in embolization of the cerebral circulation and cerebrovascular accident. 11 Although we expect vascular events have findings in orbit or brain MRI, the lack of imaging findings in our patient or the previous cases cannot rule out this mechanism.

Our patient had a history of lips filler injections. In similar patients, another mechanism that has been suggested is hypersensitivity reactions.

The immune system becomes sensitive to the filler material for any reason in the initial injection. In the subsequent injections, the immune system causes systemic or localized complications by provoking inflammation. 12 This mechanism is not acceptable in the present case for two reasons. First, the patient initially had an injection in the right temple area with no complaints, and only on the left side injection did the ocular symptoms and pain begin. Second, no skin lesions were observed at the injection site, which also weakens the concept of the inflammatory mechanism.

A retrospective study of 21 patients on the prognosis of ophthalmoplegia following filler injection for cosmetic purposes showed that ocular movements recovered entirely in 77% of patients who had diplopia at initial presentation, with incomplete recovery in 23%. 13 In the present case, the patient's diplopia and ocular movement disorder have completely vanished after 10 weeks, and only mild hypotropia and ptosis of the left eye remained.

Tips have been noted to preclude complications caused by the mechanism of vascular occlusive events (retrograde followed by anterograde): awareness of anatomical danger zones and considering the most suitable technique, 8 injection with a blunt cannula, aspiration before injection, slow retrograde injections, avoiding bolus injections greater than 0.1 mL, 2 minimal use of epinephrine, and single bolus injection. 5

Regarding the complications of vascular occlusion accidents, all studies have pointed to starting treatment as soon as possible to prevent lifelong sequels. The sooner treatment is started, the better the final prognosis. The proposed golden time is currently less than 90 minutes. 14 In a cross-sectional study by Goodman et al, hyaluronidase, warm compress, massage, glyceryl nitrate, hyperbaric oxygen, aspirin, and heparin have been shown to have the highest response rates, respectively. 15 Prado and Rodríguez-Feliz have suggested a treatment algorithm based on previous studies, including timolol eye drop, aspirin, hyaluronidase, eye globe massage, acetazolamide, neurologic consult, and anticoagulant if needed. 16 However, all studies on the effect of treatment methods have a low level of evidence; in the future, randomized clinical trial studies seem necessary to develop a treatment protocol.

In conclusion, isolated ophthalmoplegia following cosmetic filler injection is a rare complication, mostly occurring after injection to the glabella, nasolabial fold, periorbital, and lateral nasal site. This case report shows that ophthalmoplegia can also happen with temple region filler injections. Therefore, to avoid such complications, awareness of prevention techniques and available treatments are necessary when performing soft tissue fillers for gaunt appearance correction.
